# Nanomaterials for reversion of multidrug resistance in cancer: a new hope for an old idea?

**DOI:** 10.3389/fphar.2013.00134

**Published:** 2013-10-25

**Authors:** João Conde, Jesús M. de la Fuente, Pedro V. Baptista

**Affiliations:** ^1^Instituto de Nanociencia de Aragon, Universidad de ZaragozaZaragoza, Spain; ^2^Departamento de Ciências da Vida, Faculdade de Ciências e Tecnologia, CIGMH, Universidade Nova de Lisboa, Campus de CaparicaCaparica, Portugal

**Keywords:** nanoparticles, nanomaterials, multidrug resistance, cancer, drug delivery, siRNAs

Cancer is a major cause of mortality in the modern world, with more than 10 million new cases every year. This outline is expected to rise in the next few decades since the majority (59%) of people diagnosed with cancer is aged over 65. In fact, around one in three people will be diagnosed with cancer throughout their lifetime (Siegel et al., 2012; Bosetti et al., 2013).

The foundation of cancer treatment is surgery, chemotherapy, radiation, antibody-blocking therapy, or a combination of these therapies (Hanahan and Weinberg, 2000). Still, many clinical chemotherapeutic and radiotherapeutic regimes are not exceptionally effective, due to multidrug resistance mechanisms, depending on the patient and the type of tumor. Therefore, there is an urgent need for more effective and valuable cancer therapeutics, in order to reduce the impact of the chemotherapeutic agents on the healthy tissues by creating more selective systems toward the cancerous cells (Alison, 2001; Perez-Tomas, 2006).

Multi-drug resistance (MDR) in cancer refers to the capacity of cancer cells to survive or become resistant from treatment of a wide variety of drugs. Cancer chemotherapy has become progressively sophisticated within the last years; however there are not any cancer therapies 100% effective against disseminated cancer. This is in fact a major problem once approximately 70% of patients do not respond to initial chemotherapy and the five-year survival rate for these patients is a low 10–30%. Relapse is also frequent (Diseases, 2000).

Mechanisms of MDR include decreased uptake of drugs, reduced intracellular drug concentration by activation of the efflux transporters, modifications in cellular pathways by altering cell cycle checkpoints, increased metabolism of drugs, induced emergency response genes to impair apoptotic pathways and altered DNA repair mechanisms (Gottesman, 2002). P-glycoprotein (P-gp) is the best known membrane transporter used in MDR and has been first described in the late 1970s (Juliano and Ling, 1976). Since then, the phenomenon of cancer drug resistance became a hotspot of cancer research (Gottesman, 2002; Ullah, 2008).

Despite of the discovery of multiple new gene/protein expression signatures or factors associated with drug resistance by high throughput “-omics” technologies, none of these findings has been useful in producing efficient and specific diagnostic assays or for improvement of updated chemosensitizers. Clinical success has also been limited due to issues regarding safety, once one of the most common strategies against MDR is the development of ATP-binding cassette (ABC) transporter inhibitors, which are poorly effective and specific, increasing the toxicity associated with chemotherapy (Lage, 2008).

Nanotechnology and nanomaterials in particular, are expected to provide a range of devices to treat cancer as their sizes are well matched in size to biologic molecules and structures found inside living cells (Conde et al., 2012).

The development of nanoscale devices and structures has provided major breakthroughs in monitoring and fighting cancer (Qian et al., 2008; Ren et al., 2012; Conde et al., 2013). Cancer nanotechnology offers a wealth of safety and innovative tools to treat and diagnose cancer, such as multifunctional, targeted devices capable of bypassing crucial biological barriers and to deliver multiple therapeutic agents directly to cancer cells and adjacent tissues around tumor microenvironment (Sanvicens and Marco, 2008).

Nanoparticles (NPs) are usually produced to deliver and enhance the drug concentration inside the cancer cells, using both active and passive targeting. (NPs) are excellent tumor-targeting vehicles because of the unique inherent property of solid tumors. Numerous tumors present with defective vasculature and poor lymphatic drainage, due to their rapid growth, resulting in an enhanced permeability and retention (EPR) effect. This effect allows (NPs) to accumulate preferably at the tumor site. Once the tumor is directly connected to the main blood circulation system, multifunctional (NPs) may exploit several characteristics of the newly formed vasculature and efficiently target tumors (Conde et al., 2012; Schroeder et al., 2012). This effect constitutes one of the major advantages of (NPs) against MDR mechanisms. In fact, lipid (NPs) and nanocapsules, polymeric (NPs), metal (NPs), dendrimers and liposomes have been reported to circumvent drug resistance (Dong and Mumper, 2010) (Figure 1).

**Figure 1:**
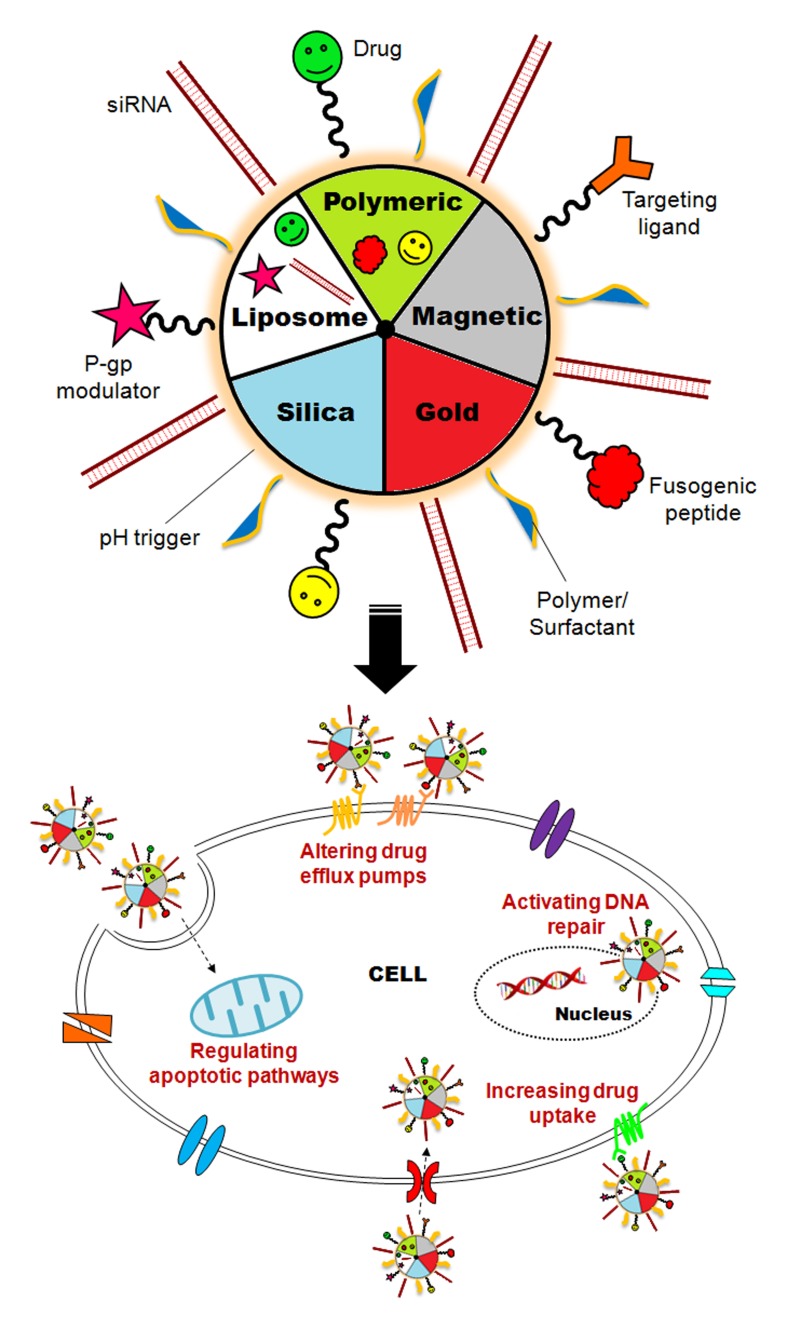
**Nanotechnology against multidrug resistance.** Smart nanomaterials (i.e., liposomes, polymeric, magnetic, silica, and gold nanoparticles functionalized with MDR inhibitors and targeting moieties) can be used to circumvent essential MDR mechanisms, by increasing drug efflux and metabolism and activating DNA repair and apoptotic pathways.

The most common nanomaterials to use against (P-gp) and ABC transporters resistance are non-ionic surfactants (i.e., poly(ethylene glycol), Tween 80® and Pluronics®) that usually form hydrogel bonds with the protein to escape from the recognition and therefore increase the uptake of the nanoformulated drug (Gao et al., 2012). The ABC transporters are also expressed in normal cells and so it is necessary to inhibit these drug efflux transporters in tumor tissues preferentially, while minimizing the inhibition in normal tissues (Patel et al., 2013).

Some of these nanomaterials have been used to overcome drug efflux by drug transporters, increasing the drug retention effect in cancer cells. These systems comprise the nanoformultation (specially with liposomes, nanodiamonds, mesoporous silica nanoparticles) of siRNAs and drugs like doxorubicin (DOX), used to hamper cancer progression, inhibiting the drug detoxification, by suppressing cell defense mechanisms, activating apoptosis and DNA repair (Minko et al., 1999; Chen et al., 2009, 2010; Chow et al., 2011).

Other drugs like vincristine, verapamil, cyclosporin, paclitaxel, oxaliplatin, cisplatin, and curcumin have been delivered using polymeric (NPs) and nanoemulsions made of surfactants (Soma et al., 2000; Devalapally et al., 2007; van Vlerken et al., 2007; Abu Lila et al., 2009; Ganta and Amiji, 2009; Song et al., 2009; Yadav et al., 2009; Aryal et al., 2010).

This combinatorial delivery of multiple drugs and MDR inhibitors opens up a huge amount of schemes to target the comprehensive mechanisms of MDR especially via the EPR effect, and at the same time it lifts up some important concerns about this approach.

Although most of these studies reported a co-delivery of multiple agents in the same nanoparticle such as siRNAs, chemotherapeutic drugs, antibodies and other MDR inhibitors, these formulations are far from achieving excellent results. This happens once the majority of drugs such as paclitaxel are hydrophobic and the MDR inhibitors like siRNAs anti-MDR associated genes are hydrophilic. This fact impairs one of the most amazing characteristic of the nanoparticle systems that is the high loading capability in the same formulation. Another problem with the loading of multiple drugs in the same nanoformulation is the molar ratios between molecules that are often incomparable and sometimes very difficult to control and quantify. This is a key element in dosage optimization and maximization of the combinatorial effects along with the nanoformulated composites (Creixell and Peppas, 2012; Gao et al., 2012).

Moreover, the interaction between chemotherapeutic drugs and MDR inhibitors (i.e., siRNAs) may occur when using coadministration. In fact, some of the drugs can strongly influence endosomal escape and siRNA recognition by the RNA Induced Silencing Complex (RISC), which are crucial obstacles/limitations for translation of RNAi into clinical setting (Lee et al., 2013). This mutual interactions between drugs and siRNAs need to be further explored and optimized.

This raises another challenge, which is the endosomal escaping of the nanoformulated drugs. Upon endocytosis the NPs-drugs usually go to the lysosomal and endosomal compartments, facing a strong acidic and enzymatic environment (Hu and Zhang, 2009). A biomolecule that bypasses the endosome would greatly increase the therapeutic effect. For example, a fusogenic peptide would promote endosomal escape by a pH-responsive mechanism in which the peptide becomes protonated at the acidic pH of the endosome, destabilizing the endosomal membrane when the protonated peptide fuses with it, enabling the delivery of the siRNAs to the cytoplasm without suffering degradation. Poly(ethylene imine) (PEI) is a cationic polymer and is also used as carrier for siRNA due to its high siRNA binding capacity and its proton sponge effect for endosomal escape (Creixell and Peppas, 2012).

However, some effective nanoparticle formulations (in particular liposomes and micelles) have been reported using the endocytic uptake of drug to avoid MDR (Thierry et al., 1993; Huwyler et al., 2002; Rapoport et al., 2002). This occurs since formulated/encapsulated drugs are more effective than free drugs, as sometimes they have more cytotoxicity to resistant cancer cells and/or improved internalization yields (Huwyler et al., 2002; Gabizon et al., 2003).

An alternative to the use of siRNA-NPs are the antisense oligodeoxynucleotides nanoformulations, once are more stable in biological matrices and have more resistance to enzymatic degradation (Conde et al., 2010; Wang et al., 2010).

In addition of active targeting, the long circulating half-life and drug release kinetics are important aspects of an effective strategy to combat MDR. In fact, the rate of drug release from the NP decides their therapeutic effectiveness. When fast drug release may lead to their lost while in blood stream circulation, a slow kinetic may predispose cancer cells to more resistance once they may not compete with the drug efflux pumps (Hu and Zhang, 2009).

To increase life-time, long circulating-NPs are usually coated with poly(ethylene glycol) (PEG), which has the ability to circulate for a prolonged period of time to allow efficient target of a particular cell/tissue/organ and retards the uptake by the macrophages and monocytes, from the reticuloendothelial system (RES) (Kommareddy et al., 2005; Uchida et al., 2005).

Concerning the stimuli-responsive drug release, pH-sensitive triggers are the most common strategies once tumoral tissues are well known for presenting acidic conditions, as well as the lysosomal and endosomal compartments, which represent a strong advantage for selective drug release. Several nanoformulations of pH-triggered drug release have been reported, especially those using pH-sensitive liposomes (Fattal et al., 2004), polymeric (NPs) (Roux et al., 2002), micelles (Lee et al., 2008) and hydrogels (Griset et al., 2009). Generally, (NPs) are coated with biomolecules (i.e., phosphatidylethanolamine, poly(ethylene glycol), poly(L-histidine), poly(L-lactic acid), polymethacrylates, poly(amidoamine)) that, when protonated, destabilize the formulation (that become leaky in acidic environment) leading to the accelerated/controlled intracellular drug release. This kinetic release overwhelms the (P-gp) drug efflux pumps (Hu and Zhang, 2009).

Although the use of organic (NPs) such as liposomes, lipids, micelles, and polymeric (NPs) constitute the major strategy to deliver high amounts of drugs and MDR inhibitors, the use of inorganic (NPs) to reverse MDR in cancer has also been reported.

The most frequent systems usually combine silica (Rigby, 2007; Chen et al., 2009), magnetic (Cheng et al., 2011; Singh et al., 2011; Klostergaard and Seeney, 2012) or gold (NPs) (Dreaden et al., 2012; Tomuleasa et al., 2012) with specific drugs and siRNAs. The silica (NPs) are ideal candidates for the loading of large amounts of drugs and other components, due to high surface area to volume ratio and large pore volume. The magnetic (NPs) allow for physical/magnetic enhancement of the passive mechanisms for the accumulation of magnetic-responsive (NPs) into tumor tissue, leading to increase cellular uptake. Gold (NPs) also have shape/size-dependent optoelectronic properties and the endosomal-based route for gold nanoparticle cellular uptake is one of the main advantages for overcome MDR (Ayers and Nasti, 2012; Creixell and Peppas, 2012).

As described above, an extensive knowledge has been gathered in the emerging field of nanomaterials to overcome multidrug resistance mechanisms. Many of the drugs available to circumvent MDR were in the past unavailable to target this mechanism due to low solubility and/or stability. Nanomaterials made these drugs a possible strategy to target MDR. However, many anti-cancer drugs have never been used in nanomedicine (Dong and Mumper, 2010). Moreover, nanomaterials have also provided an effective platform to deliver high loads of drugs (thus lower chances of resistance) in a specific and controlled way (using pH-responsive and stimuli-sensitive), with surface-modified to improve circulation time, preventing the uptake by the RES. Another advantage of using nanomaterials for MDR regression is the drastic reduction of the IC(50) value for most of the nanoformulated drugs, reducing the clinical doses of the conventional chemotherapeutic agents, which show high levels of cytotoxicity. This allows the expansion of the anticancer therapeutic window.

However, many challenges involved in biocompatibility and specificity of nanomaterials against MDR need to be further investigated. Other crucial issues embrace the fact that agents that can reduce MDR *in vitro*, are sometimes useless in patients, probably because some drugs get entrapped in circulation by serum proteins, for example. In addition, new nanoparticle-based targeting moieties such as antibodies, minibodies and peptides should be explored as an additive effect of EPR.

Moreover, it is important to realize that cancer cells need to lose their chemoprotective features mediated by MDR genes, at the same time the chemotherapy-sensitive non-cancerous cells (i.e., bone marrow stem cells) need to be protected from the effects of chemotherapeutic agents. In fact, the destruction of these cells constitutes the single most important dose-limiting toxicity factor in cancer therapy. If we could repopulate the blood system with chemoresistant blood cells the patients could receive higher doses of anticancer agents than could be given normally.

This raises the important issue of the specificity and toxicity of nanomaterials as one of the major obstacles to treat multidrug-resistant tumors. Therefore, any nanoformulation that target MDR must do so in a way that is tumoral specific, in order not to affect the normal function of healthy cells. This may be achieved using, for example, effective targeting ligands combined with valuable pro-drugs.

Some of the described drug nanoformulations here are now in human clinical trials. So it is therefore predictable that nanomaterials against MDR will eventually become commonplace in the oncology clinic in the near future.

## Author contributions

João Conde conceptualized the manuscript and wrote the draft. Pedro V. Baptista and Jesús M. de la Fuente revised the manuscript. All authors contributed in the revision process. All authors read and approved the final manuscript.

### Conflict of interest statement

The authors declare that the research was conducted in the absence of any commercial or financial relationships that could be construed as a potential conflict of interest.
